# Efficacy of Tripterygium glycosides in immune-mediated kidney diseases as a immunomodulation drug in combination with conventional immunosuppressive agents: a systematic review and meta-analysis of randomized controlled trials

**DOI:** 10.3389/fphar.2025.1525482

**Published:** 2025-07-11

**Authors:** Yaotan Li, Jinyi Hou, Xiaochang Wu, Chang Liu, Mengqi Zhou, Shijia Lin, Weijing Liu, Yaoxian Wang, Huijuan Zheng

**Affiliations:** ^1^ Dongzhimen Hospital Affiliated to Beijing University of Chinese Medicine, Beijing, China; ^2^ Beijing University of Chinese Medicine, Beijing, China; ^3^ Beijing Puren Hospital, Beijing, China; ^4^ Renal Research Institution of Beijing University of Chinese Medicine, Beijing, China; ^5^ Henan University of Chinese Medicine, Zhengzhou, China

**Keywords:** Tripterygium glycosides, immune-mediated kidney diseases, randomized controlled trials, immunosuppressive agents, meta-analysis

## Abstract

**Background:**

Immune-mediated kidney diseases involve the immune system attacking the kidneys, resulting in damage and dysfunction. Tripterygium glycosides (TG) are known for their strong immunosuppressive and anti-inflammatory effects and are commonly used alongside traditional immunosuppressive agents. However, evidence-based support for the combined use of these treatments in immune-mediated kidney diseases remains insufficient and requires further validation.

**Purpose:**

The aim of this study is to evaluate the efficacy and safety of TG combined with immunosuppressive agents in the treatment of immune-mediated kidney diseases.

**Study design:**

Systematic Review and Meta-Analysis of Randomized Controlled Trials (RCTs).

**Methods:**

We searched nine electronic databases for articles from 1 January 2014 to 1 June 2025. We included the RCTs comparing TG combined with immunosuppressive agents versus immunosuppressive agents alone. Meta-analysis was performed according to the Cochrane Handbook.

**Results:**

Thirty-six RCTs were included, involving 3,455 patients with various conditions such as membranous nephropathy (MN), IgA nephropathy (IgAN), primary nephrotic syndrome (PNS) and others. The combined use of TG and immunosuppressive agents differs from the use of immunosuppressive agents alone in terms of clinical efficacy (RR = 1.26; 95%CI: 1.22–1.30), improvement in serum creatinine (Cr) (SMD = −0.86; 95%CI: −1.11 to −0.61), blood urea nitrogen (BUN) (SMD = −0.68; 95%CI: −1.05 to −0.31), 24-h urinary total protein (24h-UTP) (SMD = −0.93; 95%CI: −1.13 to −0.74), and serum albumin (ALB) (SMD = 1.30; 95%CI: 1.08–1.52). However, there is no statistically significant difference in the improvement of total cholesterol (TC) (SMD = −0.62; 95%CI: −1.39 to 0.16). In terms of overall safety, the combination therapy shows a statistically significant difference compared to the use of immunosuppressive agents alone (RR = 0.72; 95%CI: 0.58–0.90), but no differences were observed in gastrointestinal issues, liver damage, leukopenia, and infections. Additionally, our analysis found that the combination therapy has a significant advantage over the use of immunosuppressive agents alone in reducing the recurrence rate (RR = 0.21; 95%CI: 0.10–0.44). In terms of mechanisms, the final results indicate that there are differences in interleukin-6 (IL-6) and C-reactive protein (CRP) levels between the two groups, while no differences were observed in interleukin-1 (IL-1) and tumor necrosis factor (TNF-α). However, treatment course, TG dosage, and sample size are important factors influencing the results.

**Conclusion:**

Our study suggests that the combination of TG with immunosuppressive agents offers more pronounced efficacy in treating immune mediated kidney diseases, without increasing the incidence of adverse reactions. However, our findings may be limited by the quality of the existing studies. High-quality RCTs are needed to provide more accurate evidence.

**Systematic Review Registration:**

https://www.crd.york.ac.uk/PROSPERO/view/CRD42023473530.

## 1 Introduction

The kidney and immune system are intricately linked, with the latter capable of inducing a spectrum of renal disorders through both direct and indirect mechanisms (Kant et al., 2022; Tecklenborg et al., 2018; Wen, 2022). Immune-mediated kidney diseases present significant clinical challenges due to their complex pathogenesis and potential for severe outcomes. These disorders include IgA nephropathy (IgAN), primary nephrotic syndrome (PNS), membranous nephropathy (MN), and various forms of glomerulonephritis (Anders et al., 2016; Anders et al., 2023; Cunard and Kelly, 2003). Characterized by immune system dysregulation, these conditions lead to persistent inflammation and progressive renal damage (Speer et al., 2022). Conventional immunosuppressive therapies have demonstrated efficacy in treating these conditions. These treatments include corticosteroids, cyclophosphamide, azathioprine, and tacrolimus. However, their long-term use is often limited by substantial adverse effects (Li et al., 2023; Zhang et al., 2023; Pani, 2013). Adverse effects from long-term immunosuppression-including increased infection risk, hepatotoxicity, nephrotoxicity, and metabolic complications-significantly impact patient quality of life and treatment adherence. As a result, there is an urgent need for therapeutic strategies that enhance efficacy while minimizing these detrimental side effects. And given the complexity of the pathogenesis of these diseases, a single therapeutic intervention is often insufficient for complete control. Moreover, the prolonged use of a single immunosuppressive agent may reduce drug efficacy and increase the risk of adverse events. Thus, there is a pressing need to explore novel methodologies that involve the combination of diverse immunosuppressive agents to achieve a synergistic effect.

Tripterygium wilfordii Hook F (TwHF), commonly known as “lei gong teng” or “thunder god vine” in traditional Chinese medicine (TCM), is renowned for its potent anti-inflammatory and immunosuppressive properties (Song et al., 2020; Chen et al., 2013). The active constituents, TG, are extracted from the plant’s root and have demonstrated significant efficacy in modulating immune responses. This makes them promising candidates for treating various autoimmune and inflammatory disorders. Pharmacological studies have shown that TG possess anti-inflammatory, anti-tumor, and immunomodulatory activities by inhibiting T cell activation and proliferation, reducing pro-inflammatory cytokine production, and inducing apoptosis in activated immune cells (Brinker et al., 2007; Lv et al., 2019). Clinically, TG have been widely used to treat rheumatoid arthritis, systemic lupus erythematosus, ankylosing spondylitis, and immune-mediated kidney diseases (Lin et al., 2020; Xing et al., 2024; Zhu et al., 2013). Combining TG with conventional immunosuppressive agents has shown potential to enhance therapeutic outcomes and minimize adverse effects associated with long-term immunosuppressive therapy (Zhang H. et al., 2021). This combination aims to achieve a synergistic effect, thereby improving patient outcomes in complex autoimmune conditions.

Previous systematic reviews and meta-analyses have extensively investigated the therapeutic potential of TG in the treatment of the kidney diseases and immune-related diseases (Zhu et al., 2013; Shi et al., 2022; Xie et al., 2022; Wu et al., 2020; Li H. F. et al., 2021; Liu et al., 2022; Hong et al., 2016; Zhou et al., 2022; Chen et al., 2023; Wang et al., 2017). Despite well-documented anti-inflammatory and immunomodulatory properties of TG, comprehensive evaluations are lacking. Specifically, there is insufficient research on TG’s synergistic effects with standard immunosuppressive treatments, overall efficacy, disease recurrence rates, and safety of combined approaches in immune-mediated kidney diseases. To address this gap, we conducted a systematic review and meta-analysis focusing specifically on the efficacy, safety, and clinical mechanisms of TG in combination with conventional immunosuppressive agents. By incorporating a broader range of studies and utilizing rigorous analytical methods, our research aims to provide a more robust and detailed understanding of the therapeutic benefits and underlying mechanisms of this combined treatment approach. Our research aims to provide a comprehensive evaluation of the combined treatment involving TG and conventional immunosuppressive agents for immune-mediated kidney diseases. Specifically, we focus on evaluating the overall therapeutic efficacy of this combination in treating immune-mediated kidney diseases, with particular attention to its effects on proteinuria, disease recurrence rates, and safety. Through incorporation of diverse studies and rigorous analytical methods, this meta-analysis and systematic review seeks to deliver high-quality evidence that can enhance clinical decision-making and optimize treatment strategies for patients with immune-mediated kidney diseases.

## 2 Methods

### 2.1 Materials and methods

This systematic review was conducted following a prespecified protocol, which is registered in PROSPERO under registration number CRD 42023473530. The review was conducted in accordance with the guidelines outlined in the Preferred Reporting Items for Systematic Reviews and Meta-Analyses (PRISMA) harms checklist (Zorzela et al., 2016).

### 2.2 Search strategies

We conducted a comprehensive literature search across multiple databases, including PubMed, Embase, Web of Science, ClinicalTrials.gov, China National Knowledge Infrastructure (CNKI), Wanfang Med Database, SinoMed Database, Chinese VIP Information Database, Chinese Clinical Trial Registry. The search spanned from 1 January 2014 to 1 June 2025 for each database. The search strategy employed both Medical Subject Heading (MeSH) terms and free-text terms to maximize the retrieval of relevant studies. The following search terms were used: (“Tripterygium” OR “lei gong teng” OR “thunder god vine”) AND (“immune-mediated kidney disease” OR “glomerulonephritis” OR “IgA nephropathy” OR “lupus nephritis” OR “nephrotic syndrome” OR “membranous nephropathy” OR “anti-glomerular basement membrane” OR “anti-neutrophil cytoplasmic antibody-associated vasculitis”) AND random*. To ensure comprehensive identification of randomized controlled trials, we employed the standardized RCT search filter developed by the University of Alberta: randomized controlled trial [pt] OR controlled clinical trial [pt] OR randomized [tiab] OR placebo [tiab] OR clinical trials as topic [mesh:noexp] OR randomly [tiab] OR trial [ti] NOT (animals [mh] NOT humans [mh]). The search was designed to identify RCTs evaluating the efficacy of TG in combination with conventional immunosuppressive agents (Moher et al., 2009).

### 2.3 Inclusion criteria

#### 2.3.1 Type of study

Included in this study were RCTs with parallel designs assessing the efficacy of TG in combination with conventional immunosuppressive agents for the treatment of immune-mediated kidney diseases.

#### 2.3.2 Type of participants

Participants diagnosed definitely as immune-mediated kidney diseases including MN, IgAN, LN, anti-glomerular basement membrane (anti GBM) disease, anti-neutrophil cytoplasmic antibody-associated vasculitis (AAVs) and other primary nephrotic syndrome (NS) were included.

#### 2.3.3 Types of the control group

Patients in the control group should receive conventional immunosuppressive agents treatment orally.

#### 2.3.4 Types of interventions

The intervention group should be administered with equivalent conventional immunosuppressive treatments as the control group, with matching specifications in terms of category, dosage, and treatment course, while concurrently receiving TG via oral administration over the same duration. The dosage of TG had no restrictions, and its treatment duration was the same as immunosuppressive agents.

### 2.4 Exclusion criteria

Studies will be excluded if they meet any of the following criteria: 1) non-RCTs, such as observational studies, retrospective studies, cohort studies, or case reports; 2) interventions that do not involve the use of TG or do not combine them with conventional immunosuppressive agents; 3) studies including participants with other kidney diseases, cancer, active infections, fever, coagulation abnormalities, kidney transplantation, severe liver disease, or severe cardiopulmonary disease; 4) studies with incomplete or erroneous data; 5) studies with inadequate information about intervention methods or those where results cannot be extracted; 6) duplicate studies reporting the same results.

### 2.5 Outcomes measures

The primary efficacy outcome measures include the overall response rate and renal function-related indicators such as Cr, BUN, 24h-UTP, ALB, TC and recurrence rate.

The primary safety outcome measures are adverse events (AEs) such as liver injury (ALT or AST elevation >2 times the upper limit of normal), leukopenia (blood cell count <3.0 × 10^9^/L), gastrointestinal dysfunction, and infection.

Mechanism-related indicators associated with efficacy include immune markers: IL-1, IL-6, TNF-α, and CRP.

### 2.6 Data selection and extraction

Two authors extracted the relevant data independently according to predetermined inclusion criteria. The data included: first author, year of publication, sample size, participant characteristics, type of subject, intervention duration, regimen of intervention and control, outcome measures, and adverse events. When disagreements occurred, two authors discussed to resolve them. If disagreements persisted, a third author was consulted and made the final decisions.

### 2.7 Quality assessment

The Cochrane Collaboration’s risk of bias tool was used to assess the methodological quality of the included studies (Sterne et al., 2019; Guyatt et al., 2008). The specific evaluation details can be found on this website https://www.riskofbias.info/. It included the following items: random sequence generation, allocation concealment, Blinding of participants and personnel, blinding of outcome assessment, incomplete outcome data, selective reporting and other bias. We evaluated each item as “low”, “unclear” or “high” by two authors, and disagreements were resolved by discussions with a third author. The certainty of the evidence for each outcome was assessed by using the Grading of Recommendations, Assessment, Development, and Evaluation (GRADE) approach.

### 2.8 Statistical analyses

The Stata 18.0 software (StataCorp, College Station, Texas, United States) and the Review Manager 5.4 software (The Cochrane Collaboration, 2020) were used for this meta-analysis. For continuous outcomes, we used standardized mean difference (SMD) and 95% confidence interval (CI); for dichotomous outcomes, risk ratio (RR) and 95% CI were used. Heterogeneity was tested by using Chi-squared and I^2^ statistics. A random effects model was applied if I^2^ > 50\% or ChiI^2^ test p < 0.1; otherwise, a fixed effects model was used.

To further explore the sources of heterogeneity, we sequentially employed regression and subgroup analyses to examine the effects of various factors on efficacy indicators. These factors include gender (male-to-female ratio >1 or <1), mean age (under 45 years or 45 years and above), TG dosage, treatment course, the number of concomitant immunosuppressive agents used, and sample size. Additionally, we assessed the results through sensitivity analysis by excluding each study one by one.

When the number of included studies reaches 10 or more, we will investigate potential publication bias utilizing funnel plots in conjunction with Egger’s and Begg’s tests. The P-value of less than 0.05 will be considered indicative of statistical significance.

## 3 Results

### 3.1 Selection of studies

**FIGURE 1:**
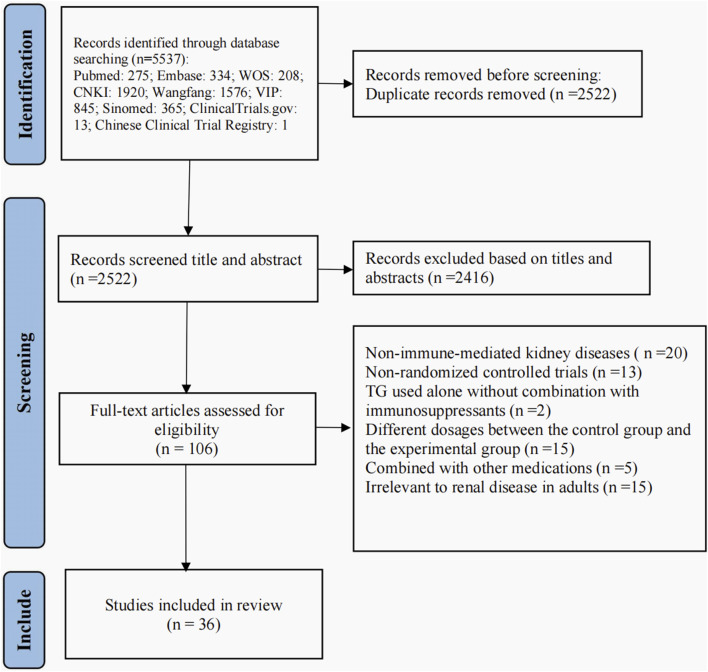
Flow diagram of trials selection.

We included 36 RCTs involving 3,455 participants in this systematic review. The study screening process and results are shown in Figure 1.

### 3.2 Description of included studies

36 RCTs (Chen, 2015; Chen et al., 2022; Chen, 2018; Cui and Li, 2020; Deng and Xie, 2014; Ding et al., 2019; Fan and Xu, 2014; Fang, 2021; Gao, 2019; Guo, 2017; Jiao et al., 2020; Lai, 2019; Li Y. et al., 2021; Li and Cao, 2022; Liu, 2017; Liu et al., 2018; Ni et al., 2022; Ni, 2017; Piao, 2015; Shen et al., 2021; Tian et al., 2017; Wang et al., 2021; Wang and Qin, 2014; Wang N. N., 2020; Wang, 2014; Wang Q., 2020; Wang, 2016; Wen, 2022; Xiong, 2019; Yan et al., 2023; Zhang, 2022; Zhang and Fan, 2022; Zhang, 2015; Zhao et al., 2018; Zheng et al., 2021; Zhou, 2016) were published in Chinese, with intervention durations ranging from 4 weeks to 48 weeks. All included RCTs had experimental groups that added TG to the immunosuppressive agents used in the control group. Among them, 19 RCTs combined TG with Prednisone Acetate Tablets, 5 RCTs combined it with Mycophenolate Mofetil (MMF), 3 RCTs combined it with Prednisone Acetate Tablets (PAT) and Tacrolimus Capsules (TAC), 3 RCTs combined it with PAT and MMF, 2 RCTs combined it with PAT and Cyclophosphamide (CTX), 1 RCT combined it with TAC, 1 RCT combined it with CTX, 1 RCT combined it with Glucocorticoids (ICS),and 1 RCT combined it with Leflunomide (lef.). The characteristics of the included RCTs are shown in Table 1.

**TABLE 1:** Details for included trials.

Study (author/year)	Sample size (Intervention/Control)	Sex (M/F)	Age (years) range or I/C mean ± SD	Study duration (weeks)	Disease type	Intervention group (regimen)	Control group (regimen)	Outcome measures
Yan et al. (2023)	118 (59/59)	70/48	44.02 ± 1.29/44.18 ± 1.36	24	PGN	TG 20 mg tid+ICS 30 mg tid	ICS 30 mg	TER, 24h-UTP, Scr, BUN, AEs, IL-6, TNF-α, hs-CRP
Zhang (2022)	80 (40/40)	58/22	47.12 ± 10.63/47.46 ± 10.52	48	IgAN	TG 20 mg tid+PAT 30 mg/d→5 mg/d	PAT 30 mg/d→5 mg/d	TER, 24h-UTP, Scr, AEs
Ni et al. (2022)	90 (45/45)	58/32	56.83 ± 7.03/57.84 ± 7.36	24	PGN	TG 90 mg/d→20 mg/d tid+PAT 30 mg/d→10 mg/d	PAT 30 mg/d→10 mg/d	TER, 24h-UTP, Scr,BUN, Alb, IL-1, IL-6, TNF-α, AEs
Li and Cao (2022)	108 (54/54)	15/93	41.6 ± 13.7/41.4 ± 13.9	24	LN	TG 90 mg/d+Lef. 20 mg/d qd	Lef.20 mg/d qd	TER, Scr, BUN,Alb, AEs
Wen (2022)	80 (40/40)	42/38	49.16 ± 5.31/48.59 ± 5.03	12	PNS	TG 30 mg/d tid+PAT60 mg/d+MMF 60 g/d→30 g/d	PAT 60 mg/d+MMF 60∼90 g/d→30 g/d	TER, Scr, Alb, 24h-UTP
Zhang and Fan (2022)	68 (34/34)	40/28	43.00 ± 10.60/40.56 ± 11.17	24	RNS	TG 60 mg/d tid+MMF 3 g/d bid→1 g/d bid	MMF 3 g/d bid→1 g/d bid	TER, 24h-UTP, Scr,BUN, RR
Chen et al. (2022)	97 (49/48)	62/35	57.01 ± 5.22/56.02 ± 5.17	24	IMN	TG 60 mg/d tid+TAC 3 mg/d bid+PAT 60 mg/d→0	TAC 3 mg/d bid+PAT 60 mg/d→0	TER, 24h-UTP, Scr, Alb, AEs
Li et al. (2021a)	107 (54/53)	68/39	42.98 ± 2.49/43.26 ± 2.58	16	IMN	TG 60 mg/d tid+TAC 0.1 mg/d bid	TAC 0.1 mg/d bid	TER, 24h-UTP, Scr, Alb, AEs
Zheng et al. (2021)	121 (61/60)	70/51	72.27 ± 3.19/72.34 ± 3.14	16	PNS	TG 60∼90 mg/d+PAT 40∼60 mg/d+CTX 120∼240 mg/d	PAT 40∼60 mg/d+CTX 120∼240 mg/d	24h-UTP, Scr,BUN, TNF-α, IL-6, hs-CRP
Fang (2021)	80 (40/40)	59/21	67.15 ± 2.27/67.25 ± 2.89	24	PNS	TG 90 mg/d tid→10 mg/d+PAT 30 mg/d qd →10 mg/d	PAT 30 mg/d qd→10 mg/d	TER, 24h-UTP, Alb, AEs
Shen et al. (2021)	104 (52/52)	41/59	53.16 ± 3.62/52.71 ± 5.12	40∼48	IgAN	TG 60 mg/d tid+PAT 60 mg/d →10 mg/d	PAT 60 mg/d →10 mg/d	TER, BUN,Alb, TC, AEs
Wang et al. (2021)	86 (43/43)	9/77	52.35 ± 8.96/51.28 ± 9.51	32	LN	TG 60 mg/d tid+CTX 0.4 g/d	CTX 0.4 g/d	TER, AEs
Jiao et al. (2020)	106 (53/53)	57/49	36.19 ± 4.82/35.48 ± 4.50	12	PNS	TG 60 mg/d tid + PAT 40–60 mg/d→30 mg/d	PAT 40∼60 mg/d→30 mg/d	TER, 24h-UTP, Alb, TNF-α, hs-CRP, IL-6
Cui and Li (2020)	60 (30/30)	29/31	48.1 ± 8.5/49.2 ± 8.4	24	IMN	TG 60 mg/d tid+PAT 60 mg/d qd+TAC 3 mg/d bid	PAT 60 mg/d qd+TAC 3 mg/d bid	TER, 24h-UTP, Scr, Alb, AEs
Wang (2020a)	104 (52/52)	62/42	55.34 ± 2.29/56.15 ± 2.14	12	RNS	TG 30 mg/d tid+PAT 16 mg/d→4∼8 mg/d+MMF 3 g/d bid	PAT 16 mg/d→4∼8 mg/d+MMF 3 g/d bid	TER, 24h-UTP, Scr, Alb
Gao (2019)	84 (42/42)	34/50	36.74 ± 6.28/37.18 ± 6.94	24	PNS	TG 60 mg/d tid+MMF 1.5 g/d bid→1.0 g/d	MMF 1.5 g/d bid→1.0 g/d	TER, 24h-UTP, Scr,BUN,Alb, AEs, RR
Lai (2019)	58 (29/29)	36/22	50.45 ± 1.83/49.12 ± 1.97	12	PNS	TG 60/d tid+PAT 30∼60 mg/d	PAT 30∼60 mg/d	TER, IL-1, TNF-α
Xiong (2019)	80 (40/40)	45/35	64.1 ± 3.5/63.2 ± 4.2	24	PNS	TG 60 mg/d tid→20 mg/d bid+PAT 30 mg/d→5 mg/d	PAT 30 mg/d→5 mg/d	TER, 24h-UTP, Scr,BUN,Alb, AEs, RR
Ding et al. (2019)	110 (55/55)	64/46	41 ± 17/41 ± 17	24	LN	TG 90 mg/d tid+MMF 20 mg/d qd+PAT 60 mg/d→10 mg/d	MMF 20 mg/d qd+PAT 60 mg/d→10 mg/d	TER, Scr, BUN, Alb, AEs
Zhao et al. (2018)	72 (36/36)	39/33	37.77 ± 5.42/37.68 ± 5.35	24	PNS	TG 60∼90 mg/d→30g∼45 mg/d+PAT 40∼60 mg/d→10∼20 mg/d	PAT 40∼60 mg/d→10∼20 mg/d	TER, 24h-UTP, Scr,BUN, Alb, TC,TG,AEs
Liu et al. (2018)	62 (31/31)	8/54	37.9 ± 3.4/36.6 ± 3.5	8	LN	TG 60 mg/d→20∼30 mg/d+PAT 60 mg/d qd	PAT 60 mg/d qd	TER, 24h-UTP, Scr, Alb, AEs
Chen (2018)	76 (38/38)	44/32	48.87 ± 8.09/49.76 ± 8.21	12	PNS	TG 60 mg/d + PAT 60 mg/d	PAT 60 mg/d	TER, 24h-UTP, Scr,BUN, Alb, IL-1β, hs-CRP
Tian et al. (2017)	280 (140/140)	151/129	72.19 ± 9.45/71.34 ± 9.28	2	PNS	TG 60 mg/d tid+PAT 60 mg/d+CTX 100 mg/d	PAT 60 mg/d+CTX 100 mg/d	TER, 24h-UTP, Alb, Scr
Liu (2017)	128 (64/64)	73/55	52.39 ± 12.37/51.82 ± 13.86	24	PNS	TG 90 mg/d + PAT 30 mg/d→10∼20 mg/d	PAT 30 mg/d→10∼20 mg/d	TER, Scr, 24h-UTP, BUN,hs-CRP, IL-6, TNF-α, AEs
Guo (2017)	170 (85/85)	88/82	35.19 ± 5.98/36.54 ± 6.07	16	PNS	TG 60 mg/d tid+MMF 1.5 g/d bid→1.0 g/d	MMF 1.5 g/d bid→1.0 g/d	TER, 24h-UTP, Scr, BUN, Alb
Ni (2017)	120 (60/60)	51/69	40.2 ± 8.3/38.5 ± 7.3	24	PNS	TG 60 mg/d tid+MMF 1.5 g/d bid→1.0 g/d	MMF 1.5 g/d bid→1.0 g/d	TER, 24h-UTP, Scr,BUN, Alb, AEs, RR
Wang (2020b)	76 (38/38)	45/31	60.5 ± 7.5	48	IMN	TG 120 mg/d→60 mg/d+TAC 3 mg/d bid+PAT 30 mg	TAC 3 mg/d bid+PAT 30 mg	TER, 24h-UTP, Scr, BUN, Alb, TC, TG, AEs
Zhou (2016)	88 (44/44)	51/37	41.36 ± 4.27/40.58 ± 4.13	48	PNS	TG 60 mg/d tid→20 mg/d+PAT 60 mg/d→20 mg/d	PAT 60 mg/d→20 mg/d	TER, 24h-UTP, AEs
Wang (2016)	80 (40/40)	47/33	34.9 ± 7.6/35.6 ± 6.4	12	PNS	TG 60 mg/d tid+PAT 60 mg/d→30 mg/d	PAT 60 mg/d→30 mg/d	TER, 24h-UTP, Alb
Piao (2015)	98 (49/49)	56/42	36.2 ± 5.4/35.5 ± 5.6	48	PNS	TG 60 mg/d tid→20 mg/d+PAT60 mg/d→20 mg/d	PAT 60 mg/d→20 mg/d	TER, 24h-UTP,BUN, Alb, Scr
Zhang (2015)	80 (40/40)	49/31	49.82 ± 11.38	24	PNS	TG 90 mg/d + PAT 30 mg/d →15 mg/d	PAT 30 mg/d →15 mg/d	TER, Scr, 24h-UTP,BUN, Alb, IL-1, TNF-α, AEs
Chen (2015)	120 (60/60)	68/52	41.6 ± 10.2	4	LN	TG 60 mg/d tid+PAT 40 mg/d qd	PAT 40 mg/d qd	TER, AEs, RRs
Fan and Xu (2014)	96 (48/48)	53/43	34.6 ± 7.4/35.2 ± 7.8	48	RNS	TG 60 mg/d tid→20 mg/d tid + PAT60 mg/d→30 mg/d	PAT 60 mg/d→30 mg/d	TER,24h-UTP, BUN,Alb, Scr, AEs
Wang (2014)	66 (33/33)	36/30	35.1 ± 2.7/34.6 ± 2.4	12	RNS	TG 60 mg/d tid→20 mg/d tid+PAT 60 mg/d →30 mg/d	PAT 60 mg/d →30 mg/d	TER, 24 h-UTP
Deng and Xie (2014)	46 (23/23)	21/25	31.6 ± 6.9/35.2 ± 8.7	8	PNS	TG 60 mg/d tid + PAT 60 mg/d	PAT 60 mg/d	TER,24 h-UTP, Alb, Scr, TC, AEs
Wang (2014)	60 (30/30)	35/25	60 ± 1.2	24	IgAN	TG 60 mg/d tid + MMF 3 g/d bid	MMF 3 g/d bid	TER

### 3.3 Risk of bias assessment

**FIGURE 2:**
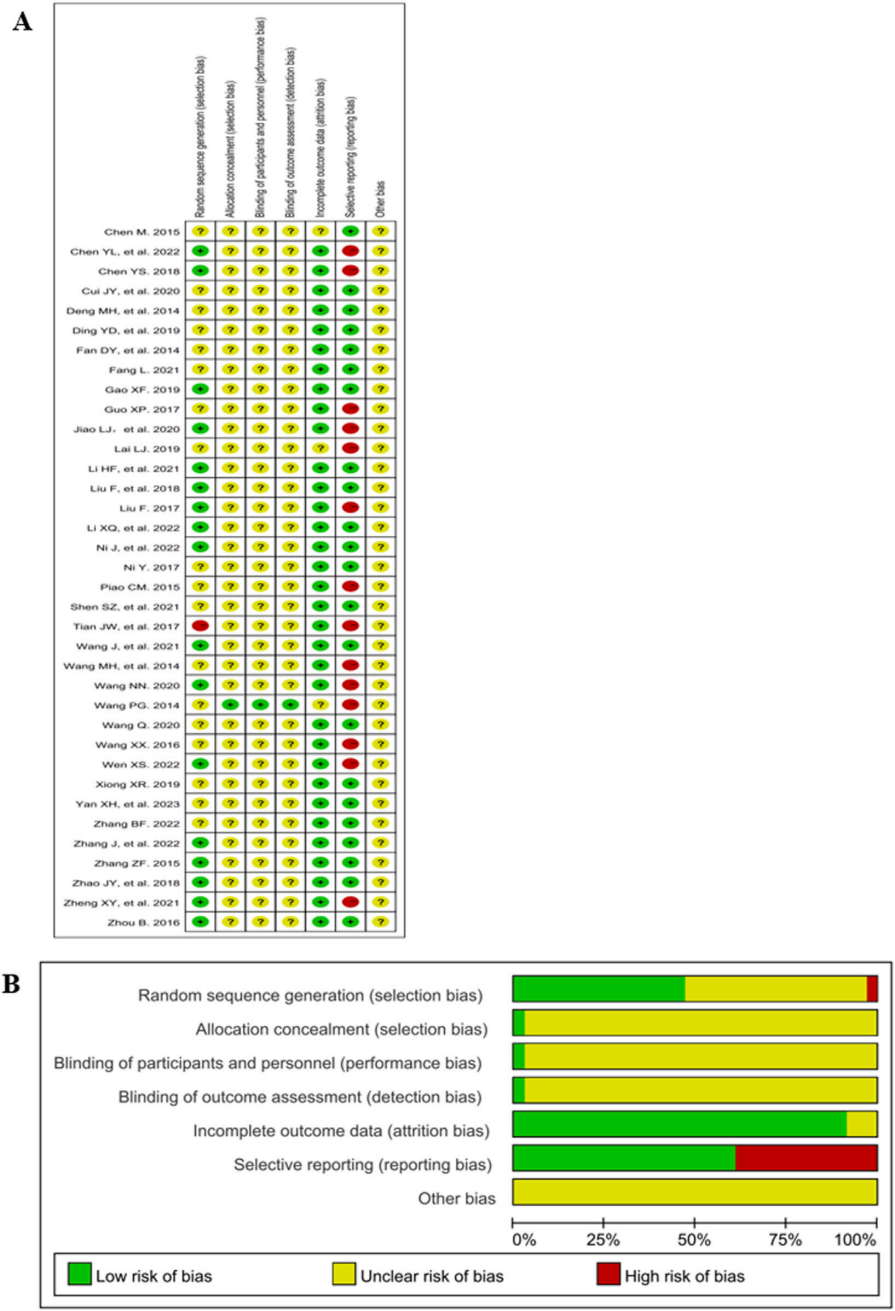
Risk of bias graph and summary. **(A)** Risk of bias graph. **(B)** Risk of bias summary.

The Cochrane risk of bias 2.0 is shown in Figure 2. 47% (17/36) of the studies mentioned a random design; and only 3% (1/36) the blinded design; 3% (1/36) described the blinding of participants, personnel and outcome assessment; 92% (33/36) described complete outcome data; 39% (14/36) (Chen et al., 2022; Chen, 2018; Guo, 2017; Jiao et al., 2020; Lai, 2019; Liu, 2017; Piao, 2015; Tian et al., 2017; Wang and Qin, 2014; Wang N. N., 2020; Wen, 2022; Zheng et al., 2021) did not report the AEs and thus were included in the studies of selective reporting.

### 3.4 Result of efficacy

#### 3.4.1 Efficacy rate

**FIGURE 3:**
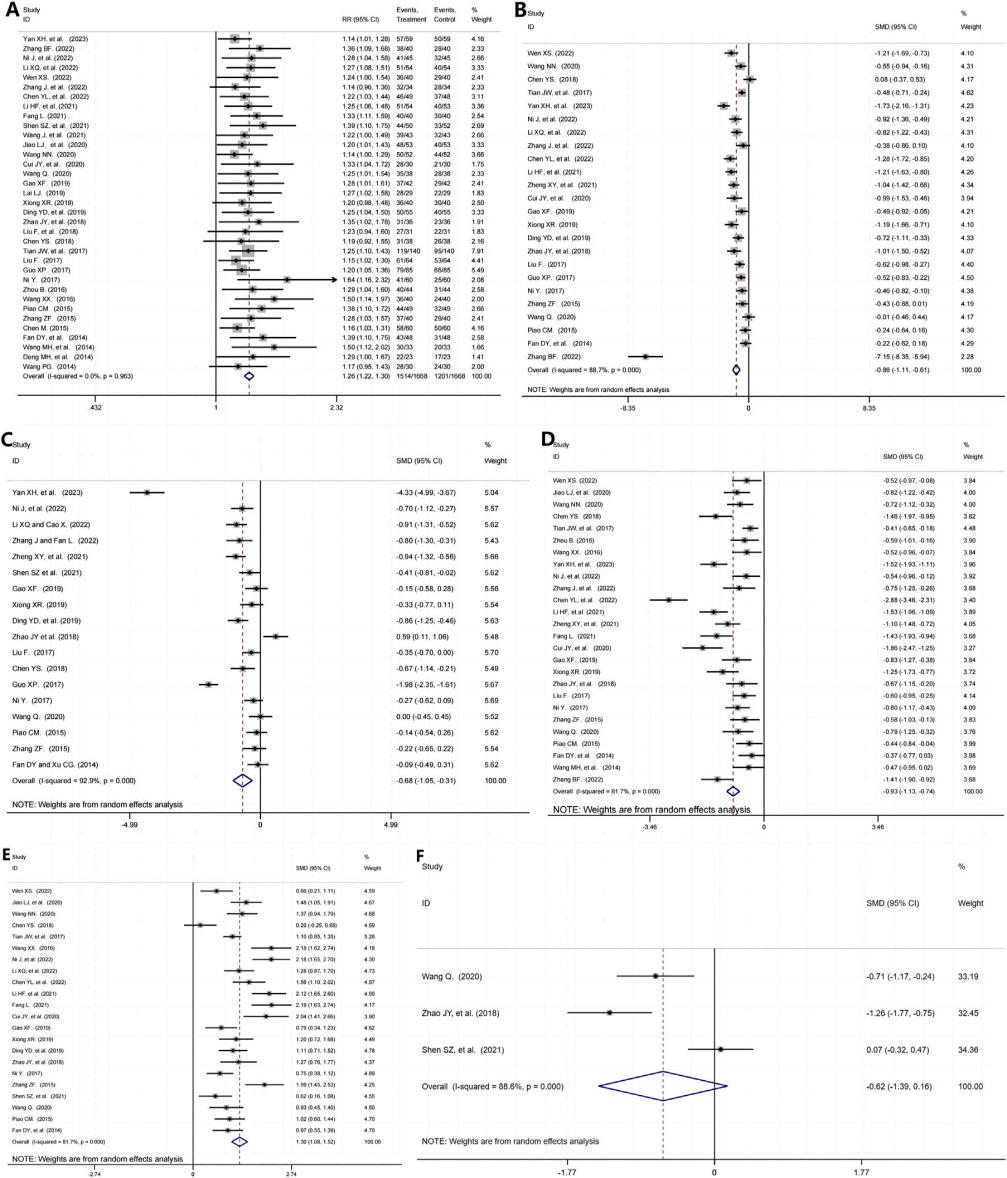
The efficacy of TG and immunosuppressants in the treatment of immune-related kidney diseases. **(A)** The overall efficacy Rate; **(B)** Cr; **(C)** BUN; **(D)** 24h-UTP; **(E)** ALB; **(F)** TC.

35 RCTs (Wen, 2022; Jiao et al., 2020; Wang N. N., 2020; Lai, 2019; Chen, 2018; Tian et al., 2017; Zhou, 2016; Wang, 2016; Chen, 2015; Deng and Xie, 2014; Yan et al., 2023; Ni et al., 2022; Li and Cao, 2022; Zhang and Fan, 2022; Chen et al., 2022; Li Y. et al., 2021; Fang, 2021; Cui and Li, 2020; Gao, 2019; Xiong, 2019; Ding et al., 2019; Zhao et al., 2018; Liu, 2017; Guo, 2017; Ni, 2017; Zhang, 2015; Wang, 2014; Piao, 2015; Fan and Xu, 2014; Wang and Qin, 2014; Shen et al., 2021; Wang et al., 2021; Wang Q., 2020; Liu et al., 2018), including 3,334 patients reported the efficacy rate. There are a significant improvement in the efficacy rate in the TG+immunosuppressive agents group compared with the immunosuppressive agents group (RR = 1.26, 95%CI: 1.22,1.30, P = 0.000, Figure 3A).

#### 3.4.2 Cr

24 studies (Wen, 2022; Wang N. N., 2020; Chen, 2018; Tian et al., 2017; Yan et al., 2023; Ni et al., 2022; Li and Cao, 2022; Zhang and Fan, 2022; Chen et al., 2022; Li Y. et al., 2021; Zheng et al., 2021; Cui and Li, 2020; Gao, 2019; Xiong, 2019; Ding et al., 2019; Zhao et al., 2018; Liu, 2017; Guo, 2017; Ni, 2017; Zhang, 2015; Wang Q., 2020; Piao, 2015; Fan and Xu, 2014; Zhang, 2022) presented results for Cr containing 2,503 participants. TG+immunosuppressive agents observed a significant reduction in Cr compared with immunosuppressive agents (SMD = -0.86, 95%CI: −1.11, −0.61, P = 0.000). For the high heterogeneity shown in Figure 3B (I^2^ = 88.7%, P = 0.000), we further investigated the potential sources of heterogeneity through meta-regression and subgroup analysis. Our findings indicated that treatment duration, TG dosage, and publication year were the primary contributors to the observed variability (Tables 2, 3).

**TABLE 2:** Subgroup analyses.

Variable	Cr				BUN				24h-UTP				ALB			
	Number of studies	WMD (95% CI)	I^2^ value (%)	P Value for heterogeneity	Number of studies	WMD (95% CI)	I^2^ value (%)	P Value for heterogeneity	Number of studies	WMD (95% CI)	I^2^ value (%)	P Value for heterogeneity	Number of studies	WMD (95%CI)	I^2^ value (%)	P Value for heterogeneity
1. Average age																
Including people <45	11	−0.70 (−0.97, −0.44)	78.7	0.000	10	−0.88 (−1.54, −0.22)	95.9	0.000	11	−0.8 (−1.04, −0.57)	69.8	0.000	9	1.19 (0.92, 1.45)	70.4	0.001
All of them ≥45	13	−1.04 (−1.47, 0.62)	92.3	0.000	8	−0.46 (−0.67, −0.25)	51.2	0.045	15	−1.04 (−1.34, −0.75)	86.3	0.000	13	1.38 (1.04, 1.72)	86.5	0.000
2. Treatment course																
≤16weeks	4	−0.53 (−0.95, −0.11)	79.8	0.002	1	−0.67 (−1.14, −0.21)	NA	NA	7	−0.69 (−0.93, −0.45)	60.3	0.019	6	1.15 (0.69, 1.61)	86.8	0.000
16 weeks < t ≤ 24 weeks	16	−0.86 (−1.04, −0.67)	68.4	0.000	13	−0.85 (−1.34, −0.35)	94.5	0.000	14	−1.15 (−1.45, −0.85)	84.1	0.000	12	1.52 (1.21, 1.83)	80.5	0.000
>24weeks	4	−1.75 (−3.35, −0.15)	97.6	0.000	4	−0.17 (−0.37, −0.03)	0.0	0.533	5	−0.68 (−1.04, −0.32)	69.2	0.011	4	0.89 (0.67,1.12)	0.0	0.611
3. TG dosage																
20 mg	2	−4.41 (−9.72, 0.90)	98.6	0.000	1	−4.33 (−4.99, −3.67)	NA	NA	2	−1.47 (−1.79, −1.16)	0.0	0.742				
30 mg	2	−0.87 (−1.51, −0.22)	76.9	0.038					2	−0.63 (−0.93, −0.34)	0.0	0.514	2	1.02 (0.32, 1.71)	80.0	0.025
60 mg	14	−0.66 (−0.87, −0.45)	74.8	0.000	11	−0.48 (−0.87, −0.08)	90.2	0.000	17	−0.96 (−1.23, −0.69)	85.1	0.000	14	1.21 (0.94, 1.49)	82.5	0.000
90 mg	5	−0.70 (−0.88, −0.53)	0.0	0.562	5	−0.61 (−0.88, −0.34)	56.4	0.057	4	−0.77 (−1.15, −0.39)	68.7	0.022	5	1.72 (1.26, 2.19)	78.8	0.001
120 mg	1	−0.01 (−0.46, 0.44)	NA	NA	1	0.00 (−0.45, 0.45)	NA	NA	1	−0.79 (−1.25, −0.32)	NA	NA	1	0.93 (0.45, 1.40)	NA	NA
4. Concomitant drugs																
1 type	16	−0.93 (−1.30, −0.57)	91.4	0.000	15	−0.70 (−1.14, −0.26)	93.9	0.000	19	−0.86 (−1.05, −0.68)	69.9	0.000	14	1.27 (0.96, 1.59)	85.2	0.000
2 types	8	−0.77 (−1.05, −0.49)	75.2	0.000	3	−0.61 (−1.17, −0.05)	82.6	0.003	7	−1.15 (−1.71, −0.60)	92.2	0.000	8	1.33 (1.02, 1.64)	75.9	0.000
5. Sample size																
<100	14	−0.96 (−1.14, −0.52)	92.1	0.000	10	−0.35 (−0.53, −0.18)	39.6	0.094	18	−0.94 (−1.21, −0.68)	82.9	0.000	14	1.35 (1.02, 1.68)	84.7	0.000
≥100	10	−0.80 (−1.03, −0.56)	76.9	0.000	8	−0.74 (−1.09, −0.39)	95.7	0.000	8	−0.92 (−1.21, −0.63)	80.8	0.000	8	1.21 (0.93, 1.50)	77.3	0.000
6. Gender (male to female ratio)																
<1	4	−0.86 (−0.90, −0.42)	24.1	0.267	4	−0.44 (−0.63, −0.24)	63.2	0.043	3	−1.12 (−1.70, −0.54)	78.6	0.009	5	1.05 (0.64, 1.47)	77.8	0.001
>1	20	−0.91 (−1.20, −0.61)	90.5	0.000	14	−0.73 (−0.84, −0.61)	93.5	0.000	23	−0.91 (−1.12, −0.70)	82.5	0.000	17	1.37 (1.11, 1.62)	82.3	0.000

**TABLE 3:** The rasult of univariate meta-regression.

Variable	Cr				24h-UTP				BUN				ALB			
	Coefficient	SE	95% confidence interval	P value	Coefficient	SE	95% confidence interval	P value	Coefficient	SE	95% confidence interval	P value	Coefficient	SE	95% confidence interval	P value
Average age	−0.761	0.430	−1.665, 0.143	0.094	−0.301	0.217	−0.749, 0.147	0.178	0.208	0.471	−0.818, 1.235	0.666	0.113	0.300	−0.522, 0.749	0.711
Treatment course	−0.042	0.017	−0.078, −0.007	0.023	−0.003	0.008	−0.019, −0.013	0.717	0.012	0.021	−0.034, −0.059	0.572	−0.014	0.012	−0.040, 0.012	0.271
TG dosage	0.025	0.009	0.006, 0.043	0.011	0.001	0.005	−0.009, −0.011	0.824	0.022	0.012	−0.005, −0.049	0.105	0.010	0.007	−0.005, 0.025	0.185
Combination medications	0.538	0.464	−0.437, 1.513	0.261	−0.065	0.238	−0.561, 0.431	0.788	−0.302	0.650	−1.719, 1.115	0.650	−0.038	0.306	−0.687, 0.611	0.903
Sample size	−0.001	0.002	−0.004, 0.005	0.797	0.002	0.003	−0.003, 0.008	0.387	−0.020	0.009	−0.038, −0.001	0.038	−0.001	0.003	−0.007, 0.004	0.586
Gender	−0.294	0.706	−1.759, −1.171	0.682	0.211	0.354	−0.519, 0.942	0.556	−0.405	0.562	−1.597, 0.787	0.482	0.304	0.293	−0.306, −0.914	0.311

#### 3.4.3 BUN

We found 18 studies (Yan et al., 2023; Ni et al., 2022; Li and Cao, 2022; Zhang and Fan, 2022; Zheng et al., 2021; Shen et al., 2021; Gao, 2019; Xiong, 2019; Ding et al., 2019; Zhao et al., 2018; Liu, 2017; Chen, 2018; Guo, 2017; Ni, 2017; Wang Q., 2020; Piao, 2015; Zhang, 2015; Fan and Xu, 2014), including 1975 patients, which analyzed BUN in which TG+immunosuppressive agents observed a significant reduction in BUN compared with immunosuppressive agents (SMD = -0.68, 95%CI: −1.05, −0.31, P = 0.000, Figure 3C). For the high heterogeneity among the included studies (I^2^ = 92.9%, P = 0.000), we further conducted regression and subgroup analyses, revealing that the sample size was the primary source of heterogeneity (Tables 2, 3).

#### 3.4.4 24h-UTP

26 trials (Wen, 2022; Jiao et al., 2020; Wang N. N., 2020; Chen, 2018; Tian et al., 2017; Zhou, 2016; Wang, 2016; Yan et al., 2023; Ni et al., 2022; Zhang and Fan, 2022; Chen et al., 2022; Li Y. et al., 2021; Zheng et al., 2021; Fang, 2021; Cui and Li, 2020; Gao, 2019; Xiong, 2019; Zhao et al., 2018; Liu, 2017; Ni, 2017; Zhang, 2015; Wang Q., 2020; Piao, 2015; Fan and Xu, 2014; Wang and Qin, 2014; Zhang, 2022), including 2,535 patients, reported 24h-UTP in which the TG+immunosuppressive agents group was improved significantly compared with the immunosuppressive agents group (SMD = -0.93, 95%CI: −1.13, −0.74, P = 0.000, Figure 3D). To address the high heterogeneity (I^2^ = 81.7%, P = 0.000), we conducted regression and subgroup analyses, which revealed that publication year was the primary source (Tables 2, 3).

#### 3.4.5 ALB

The change in ALB was measured in 22 studies (Wen, 2022; Jiao et al., 2020; Wang N. N., 2020; Chen, 2018; Tian et al., 2017; Wang, 2016; Ni et al., 2022; Li and Cao, 2022; Chen et al., 2022; Li Y. et al., 2021; Fang, 2021; Cui and Li, 2020; Gao, 2019; Xiong, 2019; Ding et al., 2019; Zhao et al., 2018; Ni, 2017; Zhang, 2015; Shen et al., 2021; Wang Q., 2020; Piao, 2015; Fan and Xu, 2014), including 2,161 patients. The pooled estimation indicated that TG+immunosuppressive agents elevated ALB significantly (SMD = 1.30, 95%CI: 1.08,1.52, P = 0.000, Figure 3E). Despite the high heterogeneity among these studies (I^2^ = 81.7%, P = 0.000), we found through futher subgroup analysis that this variability was not observed in the subgroup of studies with interventions lasting more than 24 weeks (Tables 2, 3).

#### 3.4.6 TC

TC was assessed in three RCTs (Wang Q., 2020; Zhao et al., 2018; Shen et al., 2021), including 248 patients. However, the results of the random effects model showed no statistically significant difference between the TG+immunosuppressant group and the immunosuppressant alone group (SMD = −0.62, 95%CI: −1.39,0.16, P = 0.000; I^2^ = 88.6%, P = 0.000, Figure 3F).

### 3.5 Result of safety

#### 3.5.1 The overall safety

**FIGURE 4:**
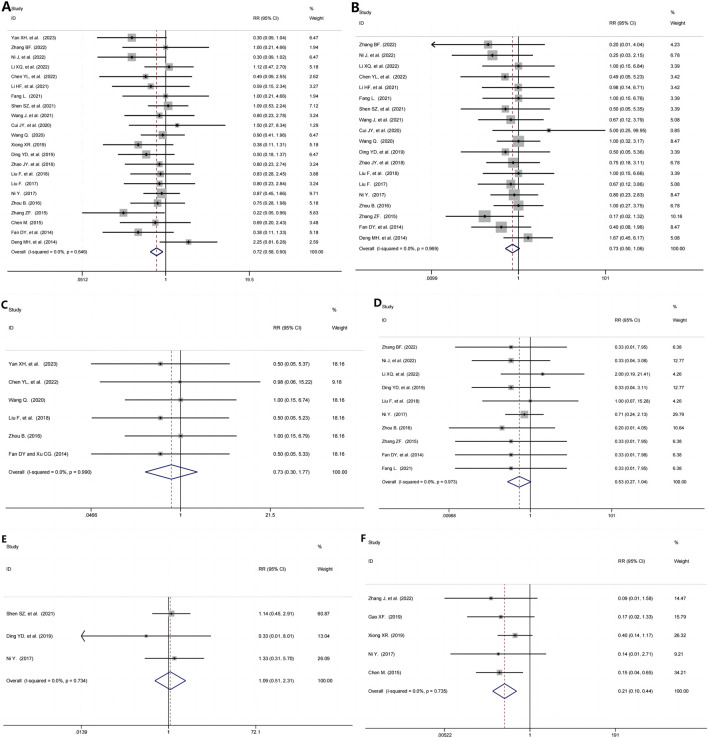
Evaluation of the Safety of TG and Immunosuppressive agents in the Treatment of Immune-mediated Kidney Diseases. **(A)** The overall safety; **(B)** The gastrointestinal adverse evets; **(C)** The liver damage; **(D)** Leukopenia; **(E)** Infection; **(F)** Recurrence rate.

22 RCTs (Yan et al., 2023; Zhang and Fan, 2022; Ni et al., 2022; Li and Cao, 2022; Chen et al., 2022; Li Y. et al., 2021; Fang, 2021; Shen et al., 2021; Wang et al., 2021; Cui and Li, 2020; Wang Q., 2020; Xiong, 2019; Ding et al., 2019; Zhao et al., 2018; Liu et al., 2018; Liu, 2017; Ni, 2017; Zhou, 2016; Zhang, 2015; Chen, 2015; Fan and Xu, 2014; Deng and Xie, 2014) evaluated the overall safety, including 1992 participants, identified some clinical significant between the TG+immunosuppressive agents group and the immunosuppressive agents group (RR = 0.72, 95%CI: 0.58, 0.90, P = 0.005; I^2^ = 0.00%, P = 0.646, Figure 4A).

#### 3.5.2 The gastrointestinal adverse evets

19 RCTs (Zhang, 2022; Ni et al., 2022; Li and Cao, 2022; Chen et al., 2022; Li Y. et al., 2021; Fang, 2021; Shen et al., 2021; Wang et al., 2021; Cui and Li, 2020; Wang Q., 2020; Ding et al., 2019; Zhao et al., 2018; Liu et al., 2018; Liu, 2017; Ni, 2017; Zhou, 2016; Zhang, 2015; Fan and Xu, 2014; Deng and Xie, 2014), including 1,690 patients, evaluated the gastrointestinal adverse events and found no statistically significant difference between TG+immunosuppressive agents group and immunosuppressive agents group (RR = 0.73, 95%CI: 0.50, 1.06, P = 0.100; I^2^ = 0.00%, P = 0.969, Figure 4B).

#### 3.5.3 The liver damage

We found that 6 studies (Yan et al., 2023; Chen et al., 2022; Wang Q., 2020; Liu et al., 2018; Zhou, 2016; Fan and Xu, 2014), including 537 patients, analyzed the liver damage in the TG+immunosuppressive agents group and immunosuppressive agents group, and no statistically significant difference was observed between the two groups (RR = 0.73, 95%CI: 0.30,1.77, P = 0.482; I^2^ = 0.00%, P = 0.990, Figure 4C).

#### 3.5.4 Leukopenia

10 studies (Zhang, 2022; Ni et al., 2022; Li and Cao, 2022; Ding et al., 2019; Liu et al., 2018; Ni, 2017; Zhou, 2016; Zhang, 2015; Fan and Xu, 2014; Fang, 2021) evaluated the safety of TG+immunosuppressive agents on leukopenia. In each of the two groups, TG+immunosuppressive agents and immunosuppressive agents, there were 914 patients. We found there was no statistically significant difference in leukopenia between the TG+immunosuppressive agents group and immunosuppressive agents group (RR = 0.53, 95%CI: 0.27,1.04, P = 0.065; I^2^ = 0.00%, P = 0.973, Figure 4D).

#### 3.5.5 Infection

The effection was assessed in 3 RCTs (Shen et al., 2021; Ding et al., 2019; Ni, 2017) with 330 participants. The pooled results indicated that the effection was no significantly by TG+immunosuppressive agents (RR = 1.09, 95%CI: 0.51,2.31, P = 0.829; I^2^ = 0.00%, P = 0.734, Figure 4E).

#### 3.5.6 Recurrence rate

5 studies (Zhang and Fan, 2022; Gao, 2019; Xiong, 2019; Ni, 2017; Chen, 2015) presented results for recurrence rate containing 472 participants. The pooled results showed that no significant between the two groups (RR = 0.21, 95%CI: 0.10, 0.44, P = 0.000; I^2^ = 0.00%, P = 0.735, Figure 4F).

### 3.6 The efficacy-related mechanism

#### 3.6.1 IL-1

**FIGURE 5:**
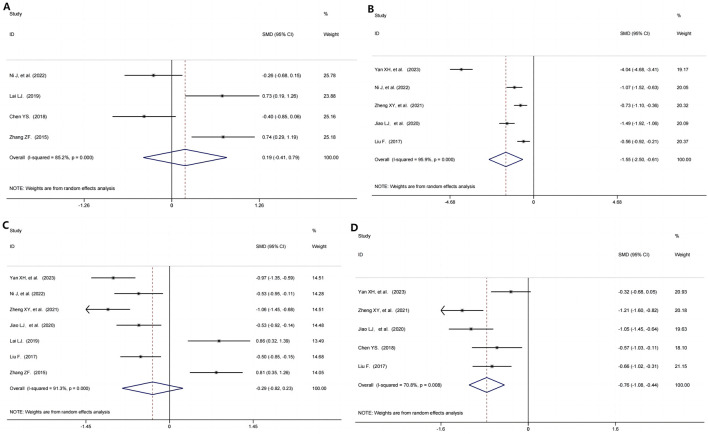
Evaluation of the efficacy-related mechanism of Immune-mediated Kidney Diseases Treated with TG and Immunosuppressive agents. **(A)** IL-1; **(B)** IL-6; **(C)** TNF-α; **(D)** CRP.

The IL-1 was assessed in 304 participants through 4 RCTs (Ni et al., 2022; Lai, 2019; Chen, 2018; Zhang, 2015). The pooled results implicated that, when comparing TG+immunosuppressive agents and immunosuppressive agents, no significant differences were shown on the IL-1 (SMD = 0.19, 95%CI: −0.41, 0.79, P = 0.530; I^2^ = 85.2%, P = 0.000, Figure 5A).

#### 3.6.2 IL-6

5 studies (Yan et al., 2023; Ni et al., 2022; Zheng et al., 2021; Jiao et al., 2020; Liu, 2017) from 563 participants evaluated the effect of TG+immunosuppressive agents on IL-6. Compared with immunosuppressive agents, TG+immunosuppressive agents significantly decreased the IL-6 (SMD = −1.55, 95%CI: −2.50, −0.61, P = 0.001, Figure 5B). There was high heterogeneity among the included studies on IL-6 (I^2^ = 95.9%, P = 0.000).

#### 3.6.3 TNF-α

A meta-analysis of 7 RCTs (Yan et al., 2023; Ni et al., 2022; Zheng et al., 2021; Jiao et al., 2020; Lai, 2019; Liu, 2017; Zhang, 2015), including 701 participants, identified no significant difference between the TG+immunosuppressive agents group and immunosuppressive agents group (SMD = −0.29, 95%CI: −0.82, 0.23, P = 0.274; I^2^ = 91.3%, P = 0.000, Figure 5C).

#### 3.6.4 CRP

5 trials (Yan et al., 2023; Zheng et al., 2021; Jiao et al., 2020; Chen, 2018; Liu, 2017) reported CRP in which the TG+immunosuppressive agents group was decreased significantly compared with the immunosuppressive agents group (SMD = −0.76, 95%CI: −1.08, −0.44, P = 0.000, Figure 5D), but with high heterogeneity (I^2^ = 70.8%, P = 0.008).

### 3.7 Publication bias and sensitivity analysis

**FIGURE 6:**
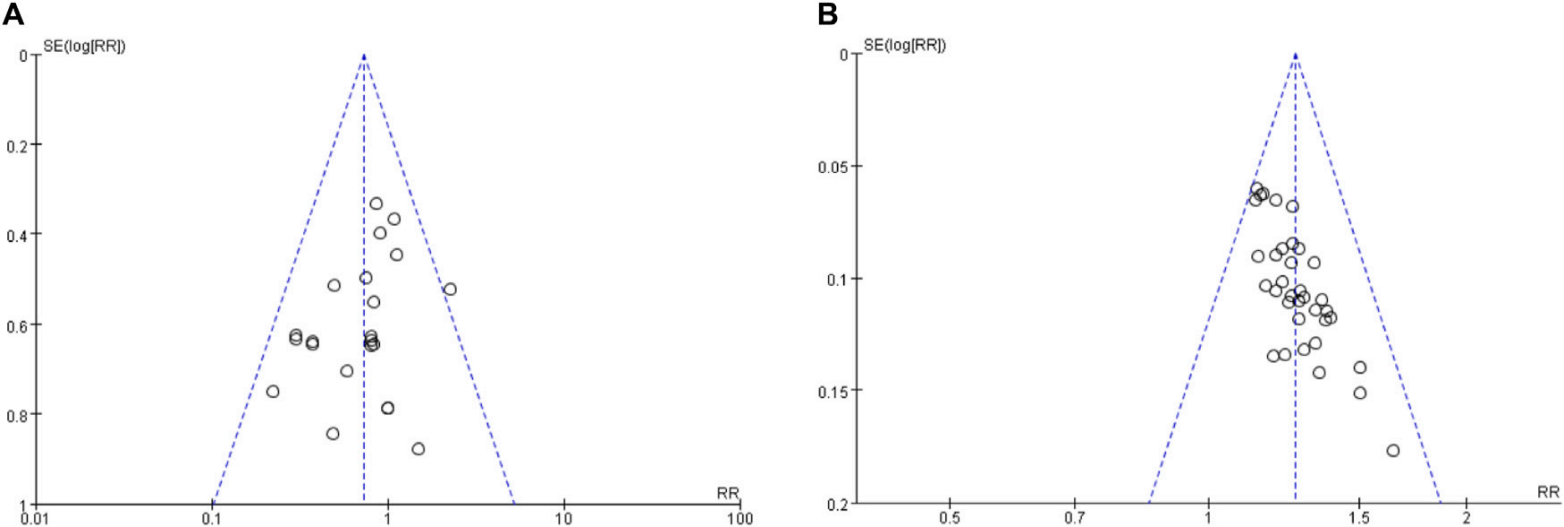
Funnel plot for publication bias assessment. **(A)** The funnel plot of overall safety; **(B)** The funnel plot of overall efficacy.

In this study, we employed funnel plots (Figure 6), Egger’s tests, and Begg’s tests to investigate potential publication bias in the included RCTs. Visual inspection of the funnel plots did not reveal any publication bias for recurrence rate and adverse events (AEs) across the studies. Furthermore, the results of Egger’s and Begg’s tests indicated no publication bias in the meta-analysis of recurrence rate (Egger’s test: p = 0.132; Begg’s test: p = 1.0000) and AEs (Egger’s test: p = 0.243; Begg’s test: p = 0.2711).

### 3.8 GRADE assessment

Considering the limitations of this study, apart from one RCT which performed random grouping based on enrollment time, the results related to efficacy rate, 24h-UTP, ALB, and Cr were downgraded. The other outcomes evaluated in the study were not downgraded. In this study, heterogeneity >50% was downgraded. In terms of indirectness, due to the consistency between PICO (Population, Intervention, Comparison, Outcome) and the research question, no downgrading was applied. Regarding imprecision, some studies were downgraded due to crossing the equivalence line and not reaching the optimal sample size. Considering the limitations of this study, and the absence of significant publication bias as indicated by the funnel plots for various outcomes, no further downgrading was applied, Table 4.

**TABLE 4:** Assessment of evidence quality.

Outcome indicators	Number of stufies	Sample size (I/C)	Limitation	Inconsistency	Imprecision	Indirectness	Publication bias	Effect size	Quality of evidence
1. Result of efficacy									
Efficacy Rate	35	1,668/1,666	−1	0	0	0	0	RR = 1.26, 95%CI (1.22∼1.30)	⊕⊕⊕⊝ MODERATE
Cr	24	1,253/1,250	−1	−2	0	0	0	SMD = −0.86, 95%CI (−1.11∼−0.61)	⊕⊝⊝⊝ VERY LOW
BUN	18	898/897	0	−2	0	0	0	SMD = −0.68, 95%CI (−1.05∼−0.31)	⊕⊕⊝⊝ LOW
24h-UTP	26	1,269/1,266	−1	−2	0	0	0	SMD = −0.93, 95%CI (−1.13∼−0.74)	⊕⊝⊝⊝ VERY LOW
ALB	22	1,093/1,091	−1	−2	0	0	0	SMD = 1.30, 95%CI (1.08∼1.52)	⊕⊝⊝⊝ VERY LOW
TC	3	124/124	0	−2	0	−1	0	SMD = −0.62, 95%CI(-1.39∼0.61)	⊕⊝⊝⊝ VERY LOW
2. Result of safety									
The overall safety	22	1,001/1,001	0	0	0	0	0	RR = 0.72, 95%CI (0.58∼0.90)	⊕⊕⊕⊕ High
The gastrointestinal adverse evets	19	843/841	0	0	0	−1	0	RR = 0.73, 95%CI (0.50∼1.06)	⊕⊕⊕⊝ MODERATE
The liver damage	7	309/308	0	0	0	−1	0	RR = 0.73, 95%CI (0.30∼1.77)	⊕⊕⊕⊝ MODERATE
Leukopenia	10	457/457	0	0	0	−1	0	RR = 0.53, 95%CI (0.27∼1.04)	⊕⊕⊕⊝ MODERATE
Infection	3	165/165	0	0	0	−2	0	RR = 1.09, 95%CI (0.51∼2.31)	⊕⊕⊝⊝ LOW
Recurrence rate	5	238/236	0	0	0	0	0	RR = 0.21, 95%CI (0.10∼0.44)	⊕⊕⊕⊕ High
3. The efficacy-related mechanism									
IL-1	4	152/152	0	−2	0	−2	0	SMD = 0.19, 95%CI (−0.41∼0.79)	⊕⊝⊝⊝ VERY LOW
IL-6	5	282/281	0	−2	0	0	0	SMD = −1.55, 95%CI (−2.50∼−0.61)	⊕⊕⊝⊝ LOW
TNF-α	7	351/350	0	−2	0	−1	0	SMD = −0.29, 95%CI (−0.82∼0.23)	⊕⊝⊝⊝ VERY LOW
CRP	5	275/274	0	−1	0	0	0	SMD = −0.76, 95%CI (−1.08∼−0.44)	⊕⊕⊕⊝ MODERATE

## 4 Discussion

Systematic reviews and meta-analyses play the critical roles in the development of medicine. They can provide a comprehensive overview of the state of knowledge in a field, helping scholars identify future research priorities. Additionally, they can address questions that individual studies are unable to answer and uncover issues present in primary research (Page et al., 2021). Immune-related kidney diseases involve immune-mediated damage to the kidneys, leading to inflammation and impairment of renal structures and function (Kronbichler et al., 2023; Suárez-Fueyo et al., 2017). These conditions can progress to chronic kidney disease if not effectively managed. This study emphasizes the combined use of TG and conventional immunosuppressive agents, aiming to systematically evaluate their overall efficacy, disease remission rates, and safety in treating these conditions. Meanwhile, our study aims to address the gaps identified in previous studies (Basso et al., 2021), specifically the lack of comprehensive evaluation regarding the synergistic effects of TG and immunosuppressive agents, and seeks to provide a more robust understanding of their combined therapeutic benefits and safety profile.

### 4.1 Efficacy evaluation

In this study, we conducted a comprehensive evaluation of the combined therapy of TG+immunosuppressive agents for treating immune-mediated kidney diseases. The results showed that this combination therapy, compared to the use of immunosuppressants alone, significantly greater improvements across several key indicators, including 24h-UTP, Cr, BUN, and ALB. TG, a traditional Chinese medicine, possesses immune-regulating and anti-inflammatory properties that may help alleviate damage and inflammation in the glomeruli (Zhang K. et al., 2021). Proteinuria, a key clinical marker of immune-related kidney diseases, is often associated with kidney disease activity and impaired glomerular filtration function (Kopp et al., 2020). Additionally, Cr and BUN levels are crucial biochemical indicators of renal function. The reduction in these markers may reflect the treatment’s effectiveness in mitigating renal inflammation and improving kidney function. Our study’s analysis demonstrates that the combination therapy of TG and immunosuppressive agents significantly reduced 24h-UTP, Cr, and BUN. This finding suggests that the combination of TG and immunosuppressive agents has notable efficacy in decreasing proteinuria, Cr and BUN.

### 4.2 Safety evaluation

Meanwhile, we also conducted a safety assessment, and the meta-analysis results indicated that the combination of TG and immunosuppressive agents was associated with a lower overall incidence of adverse events compared to immunosuppressive agents, with less variability in the results. However, for adverse events such as gastrointestinal reactions, leukopenia, and liver damage, our meta-analysis showed no significant differences between the combination therapy and the control group. This suggests that TG has a favorable safety profile in the treatment of immune-mediated kidney diseases and does not increase the risk of adverse events when used in combination with immunosuppressive agents.

### 4.3 Recurrence rate

We also investigated the recurrence rates of the disease during follow-up in the included RCTs. The results showed that the combination of TG and immunosuppressive agents was more effective in controlling disease recurrence compared to immunosuppressive agents alone, with lower heterogeneity in the results.

### 4.4 Efficacy mechanism

Understanding a drug’s mechanism is crucial for optimizing efficacy and safety, developing new drugs, personalizing treatments, predicting side effects, and advancing scientific knowledge, enhancing outcomes, and reduce adverse effects. In this study, we included immune mechanism-mediated indicators and systematically analyzed the mechanisms of action of TG in the combined therapy. We found that the combination of TG and immunosuppressive agents significantly reduced CRP and IL-6 levels, but showed no difference in IL-1 and TNF-α levels. Our findings indicate that the combination of TG and immunosuppressive agents significantly reduced IL-6 and CRP levels, but had no significant effect on IL-1 and TNF-α levels. IL-6 and CRP play crucial roles in the pathogenesis of immune-mediated kidney diseases. IL-6 is a pro-inflammatory cytokine that plays an important role in inflammatory responses and immune regulation, and its elevation is typically associated with disease activity and renal damage (Kreiner et al., 2022). CRP is an acute-phase protein, and its increased levels often reflect systemic inflammation and tissue damage (Sproston and Ashworth, 2018). Previous studies have shown that reducing IL-6 and CRP levels may help mitigate inflammatory responses and improve disease outcomes in kidney diseases (Cao et al., 2019). Large-scale RCTs are needed to further validate these findings, which will deepen our understanding of the roles and mechanisms of these inflammatory markers in immune-mediated kidney diseases and provide the foundation for developing new treatments.

### 4.5 Sources of heterogeneity and Clinical Implications

Our analysis revealed substantial heterogeneity across key renal function outcomes. Through univariate meta-regression and subgroup analyses, we identified several significant contributing factors: (1) Treatment duration: treatment course significantly influenced serum creatinine reduction (coefficient = −0.042, p = 0.023). Interestingly, the relationship between treatment duration and heterogeneity was complex: short-term studies (<16 weeks) showed I^2^ = 79.8%, medium-term studies (16–24 weeks) demonstrated the lowest heterogeneity (I^2^ = 68.4%), while long-term studies (>24 weeks) paradoxically showed the highest heterogeneity (I^2^ = 97.6%). This suggests that medium-term treatment durations may provide the most consistent therapeutic outcomes. (2) TG dosage: meta-regression revealed a paradoxical relationship where higher TG doses showed smaller effect sizes for creatinine (coefficient = 0.025, p = 0.011). Subgroup analysis demonstrated that 60 mg daily achieved optimal balance between efficacy and consistency (I^2^ = 74.8% for Cr, 85.1% for 24h-UTP), while 90 mg dosing showed no heterogeneity for creatinine (I^2^ = 0.0%) but maintained heterogeneity for proteinuria (I^2^ = 68.7%). (3) study design factors: sample size significantly influenced result stability. Larger studies (n > 100) demonstrated reduced heterogeneity compared to smaller trials across multiple outcomes: creatinine (I^2^ = 76.9% vs. 92.1%), BUN (I^2^ = 95.7% vs. 39.6%), and 24h-UTP (I^2^ = 80.8% vs. 82.9%). (4) Patient demographics: age stratification revealed differential treatment responses. Patients <45 years showed greater heterogeneity for creatinine (I^2^ = 78.7%) and 24h-UTP (I^2^ = 69.8%) compared to older patients, though older patients demonstrated higher creatinine heterogeneity (I^2^ = 92.3%). Gender distribution also influenced outcomes, with male-predominant studies (ratio >1) showing higher heterogeneity for most parameters. (5) Clinical Implications: These findings suggest that TG therapy should be administered at moderate doses (60 mg daily) for extended periods (>24 weeks) to achieve optimal therapeutic consistency and minimize treatment variability.

### 4.6 The key factors affecting the efficacy of TG+immunosuppressive agents in treating immune-mediated kidney diseases

In meta-analysis, heterogeneity is used to assess the variability between different study results and reveals inconsistencies among studies. By quantifying heterogeneity, researchers can identify and explain the sources of result differences, and investigating the impact of various factors on heterogeneity is crucial for personalized treatment in clinical practice. In our study, we found substantial heterogeneity in Cr, BUN, 24h-UTP, and ALB. To further clarify the sources of this heterogeneity, we innovatively conducted meta-regression analysis and subgroup analysis on factors such as patient age, treatment duration, TG dosage, the number of immunosuppressive agents used in combination with TG, sample size, and publication year, to investigate their impact on TG combined therapy. This research helps identify key factors contributing to treatment effect variability by exploring the impact of various factors on heterogeneity, thereby optimizing treatment plans and personalizing therapy. Understanding the sources of heterogeneity also provides direction for future studies, promoting more precise treatment strategies and improving clinical practice.

In regression analysis, we found that treatment duration and TG dosage were sources of heterogeneity in Cr. Further subgroup analysis revealed that the effect size of Cr improvement increased with longer treatment course but showed a decreasing trend with higher TG dosages. And the ample size was identified as the primary source of heterogeneity in BUN levels. Further subgroup analysis revealed that groups with larger sample sizes exhibited an increasing trend in the effect size of BUN improvement, which could be due to various factors, such as more reliable results due to increased statistical power, reduced random error, or a broader representation of patient characteristics, which may lead to a clearer demonstration of the treatment effect.

Despite conducting retrospective analysis, subgroup analysis, Egger’s test, and Begg’s test to explore the potential sources of heterogeneity, we regret that we were still unable to identify the sources of heterogeneity for 24-h UTP and ALB. This indicates that although heterogeneity exists in the included studies, the current analytical methods and statistical tests failed to reveal specific causes. This could be due to the complex interplay of various factors such as study design, interventions, or outcome measurement methods. Therefore, we recommend further exploration of potential factors that may influence treatment effects in future research, and considering the use of more methods and data to deeply analyze and interpret this heterogeneity.

### 4.7 Impact of methodological bias on study outcomes

The high risk of bias identified in our included studies has important implications for the interpretation of results. The lack of blinding in 97% of studies (35/36) is particularly concerning for outcomes such as total effective rate, which may involve subjective clinical assessments. Performance bias may have occurred as unblinded clinicians might have altered their treatment approaches, dose adjustments, or co-intervention strategies based on knowledge of group allocation. Detection bias is equally problematic, as unblinded outcome assessors may have unconsciously favored the intervention group when evaluating treatment responses, particularly for composite endpoints like “total effective rate”. The absence of allocation concealment reporting in most studies (33/36) further compounds these concerns, as it may have led to selection bias during patient enrollment. Additionally, the high proportion of studies with unclear randomization methods (19/36) raises questions about the comparability of treatment groups at baseline.

A *post hoc* sensitivity analysis was not performed in our original analysis, which represents a limitation of our study. Future meta-analyses in this field should routinely conduct sensitivity analyses excluding studies with high risk of bias (defined as unclear or high risk in≥3 bias domains) to assess the robustness of findings. Given the prevalence of methodological concerns in our included studies, such analyses would be crucial for determining whether the observed treatment benefits persist when only higher-quality studies are considered.

The consistency of effect directions across multiple outcomes (efficacy rate, renal function parameters, and inflammatory markers) provides some reassurance regarding the validity of our findings, though the magnitude of effects may be overestimated due to the methodological limitations described above.

### 4.8 Highlights and limitation

Although an increasing number of studies have shown that TG can slow the progression of CKD through mechanisms such as immunomodulation, anti-inflammation, antioxidation, and anti-fibrosis, there are few reviews and meta-analyses investigating the efficacy and safety of TG combined with immunosuppressive agents in the treatment of immune-mediated kidney diseases. Our meta-analysis is the first to comprehensively evaluate the combined use of TG and immunosuppressive agents in treating immune-mediated kidney diseases using standard meta-analysis methods. The results indicate that, compared with the use of immunosuppressive agents alone, the combination therapy has comparable therapeutic efficacy and does not increase the incidence of adverse events.

Our study also has serious limitations. (1) All 36 included RCTs were conducted exclusively in China and published in Chinese language journals. This geographic and linguistic homogeneity significantly limits the external validity and generalizability of our findings to non-Asian populations. Genetic polymorphisms in drug metabolism, differences in baseline kidney disease prevalence, and varying healthcare delivery systems may influence treatment responses across different ethnic groups. Therefore, caution should be exercised when extrapolating these results to Western or other non-Asian populations. (2) The methodological quality of included studies presents substantial concerns. Only 47% of trials (17/36) reported adequate randomization methods, with a mere 3% (1/36) implementing proper blinding of participants, personnel, or outcome assessors. Allocation concealment was largely unreported across studies. This lack of blinding is particularly problematic for subjective outcomes such as symptom assessment and may have introduced performance and detection bias, potentially overestimating treatment effects. (3) Our search strategy excluded conference proceedings, technical reports, and unpublished governmental documents. This omission may have introduced publication bias, as negative or null findings are often underrepresented in published literature. The exclusion of grey literature may have led to an overestimation of treatment benefits and underestimation of adverse events. (4) Despite comprehensive subgroup analyses and meta-regression, substantial heterogeneity (I^2^>70%) persisted in key outcomes including serum creatinine, and 24h-UTP. This unexplained variance suggests that important effect modifiers remain unidentified, limiting the precision of our pooled estimates.

## 5 Conclusion

Our meta-analysis suggests that TG combined with conventional immunosuppressive agents may improve renal function parameters, including reduced serum creatinine, blood urea nitrogen, and 24h-UTP, while increasing serum albumin levels. The combination therapy appears to maintain a favorable safety profile without increasing adverse event rates. However, these findings should be interpreted cautiously given the methodological limitations and geographic homogeneity of included studies. Well-designed, multinational randomized controlled trials with appropriate blinding are needed to confirm these preliminary findings and establish the role of TG as adjunctive therapy in immune-mediated kidney diseases.

## Data Availability

The original contributions presented in the study are included in the article/Supplementary Material, further inquiries can be directed to the corresponding authors.

## Abbreviations

ICS, Glucocorticoids; TER, Total effective rate; 24h-UTP, 24-h urinary protein; Scr, serum creatinine; AEs, incidence of adverse events; PGN, Primary glomerulonephritis; IgAN, IgA nephropathy; PAT, Prednisone Acetate Tablets; Alb, Plasma albumin; LN, Lupus Nephritis; MMF, Mycophenolate Mofetil; RR, recurrence rate; IMN, Idiopathic membranous nephropathy; TAC, Tacrolimus Capsules; HSPN, Henoch–Schonlein purpura nephritis; PNS, Primary nephrotic syndrome; CTX, Cyclophosphamide; MT, Methylprednisolone Tablets; Lef., Leflunomide; TG, Tripterygium glycosides; TCM, Traditional Chinese Medicine.
