# Supplementary material for: Efficacy of Tripterygium glycosides in immune-mediated kidney diseases as a immunomodulation drug in combination with conventional immunosuppressive agents: a systematic review and meta-analysis of randomized controlled trials
Source: Front Pharmacol. 2025 Jul 11;16:1525482. doi: 10.3389/fphar.2025.1525482 (PMC12289580; doi:10.3389/fphar.2025.1525482)
Supplement: Supplementary file 1 [file Supplementaryfile1.pdf]

1. PubMed/MEDLINE (via PubMed interface)

#1 “Tripterygium glycosides”[Mesh] OR “Tripterygium wilfordii”[Mesh]

#2 “tripterygium glycosides”[Title/Abstract] OR “tripterygium wilfordii”[Title/Abstract] OR “thunder god vine”[Title/Abstract] OR “lei gong teng”[Title/Abstract] OR “TG”[Title/Abstract] OR “TWP”[Title/Abstract]

#3 #1 OR #2

#4 “Kidney Diseases”[Mesh] OR “Glomerulonephritis”[Mesh] OR “Nephrotic Syndrome”[Mesh] OR “Nephritis”[Mesh]

#5“kidney disease*”[Title/Abstract] OR “renal disease*”[Title/Abstract] OR “glomerulonephritis”[Title/Abstract] OR “nephrotic syndrome”[Title/Abstract] OR “nephritis”[Title/Abstract] OR “proteinuria”[Title/Abstract] OR “immune-mediated kidney disease*”[Title/Abstract]

#6 #4 OR #5

#7“Immunosuppressive Agents”[Mesh] OR “Adrenal Cortex Hormones”[Mesh] OR “Mycophenolic Acid”[Mesh] OR “Tacrolimus”[Mesh]

#8“immunosuppressive”[Title/Abstract] OR “immunosuppressant*”[Title/Abstract] OR “corticosteroid*”[Title/Abstract] OR “prednisone”[Title/Abstract] OR “prednisolone”[Title/Abstract] OR “mycophenolate”[Title/Abstract] OR “tacrolimus”[Title/Abstract] OR “cyclosporine”[Title/Abstract]

#9 #7 OR #8

#10 “Randomized Controlled Trial”[Publication Type] OR “Controlled Clinical Trial”[Publication Type] OR “randomized”[Title/Abstract] OR “placebo”[Title/Abstract] OR “randomly”[Title/Abstract] OR “trial”[Title/Abstract] OR “groups”[Title/Abstract]

#11 #3 AND #6 AND #9 AND #10

2. Embase (via Ovid interface)

1. exp tripterygium glycoside/ or exp tripterygium wilfordii/
2. (tripterygium adj3 (glycoside* or wilfordii)).ti,ab,kw.
3. (thunder god vine or lei gong teng or TG or TWP).ti,ab,kw.

4. 1 or 2 or 3

5. exp kidney disease/ or exp glomerulonephritis/ or exp nephrotic syndrome/ or exp nephritis/

6. (kidney disease* or renal disease* or glomerulonephritis or nephrotic syndrome or nephritis or proteinuria).ti,ab,kw.

7. (immune adj3 mediated adj3 kidney adj3 disease*).ti,ab,kw.

8. 5 or 6 or 7

9. exp immunosuppressive agent/ or exp corticosteroid/ or exp mycophenolic acid/ or exp tacrolimus/

10. (immunosuppressive or immunosuppressant* or corticosteroid* or prednisone or prednisolone or mycophenolate or tacrolimus or cyclosporine).ti,ab,kw.

11. 9 or 10

12. exp randomized controlled trial/ or exp controlled clinical trial/

13. (randomized or randomised or placebo or randomly or trial or groups).ti,ab,kw.

14. 12 or 13

15. 4 and 8 and 11 and 14

3. Cochrane Central Register of Controlled Trials (CENTRAL)

#1 MeSH descriptor: [Tripterygium glycosides] explode all trees

#2 MeSH descriptor: [Tripterygium wilfordii] explode all trees

#3 (tripterygium near/3 (glycoside* or wilfordii)):ti,ab,kw

#4 (“thunder god vine” or “lei gong teng” or TG or TWP):ti,ab,kw

#5 #1 or #2 or #3 or #4

#6 MeSH descriptor: [Kidney Diseases] explode all trees

#7 MeSH descriptor: [Glomerulonephritis] explode all trees

#8 MeSH descriptor: [Nephrotic Syndrome] explode all trees

#9 (“kidney disease*” or “renal disease*” or glomerulonephritis or “nephrotic syndrome” or nephritis or proteinuria):ti,ab,kw

#10 (immune near/3 mediated near/3 kidney near/3 disease*):ti,ab,kw

#11 #6 or #7 or #8 or #9 or #10

#12 MeSH descriptor: [Immunosuppressive Agents] explode all trees

#13 MeSH descriptor: [Adrenal Cortex Hormones] explode all trees

#14 (immunosuppressive or immunosuppressant* or corticosteroid* or prednisone or prednisolone or mycophenolate or tacrolimus or cyclosporine):ti,ab,kw

#15 #12 or #13 or #14

#16 #5 and #11 and #15

4. Web of Science Core Collection

#1 TS=(“tripterygium glycosides” OR “tripterygium wilfordii” OR “thunder god vine” OR “lei gong teng” OR (TG AND (tripterygium OR wilfordii)) OR TWP)

#2 TS=(“kidney disease*” OR “renal disease*” OR glomerulonephritis OR “nephrotic syndrome” OR nephritis OR proteinuria OR “immune-mediated kidney disease*”)

#3 TS=(immunosuppressive OR immunosuppressant* OR corticosteroid* OR prednisone OR prednisolone OR mycophenolate OR tacrolimus OR cyclosporine)

#4 TS=(randomized OR randomised OR “controlled trial” OR placebo OR randomly OR trial OR “clinical trial”)

#5 #1 AND #2 AND #3 AND #4

5. Scopus

(TITLE-ABS-KEY (“tripterygium glycosides” OR “tripterygium wilfordii” OR “thunder god vine” OR “lei gong teng” OR (tg AND (tripterygium OR wilfordii)) OR twp)) AND (TITLE-ABS-KEY (“kidney disease*” OR “renal disease*” OR glomerulonephritis OR “nephrotic syndrome” OR nephritis OR proteinuria OR “immune-mediated kidney disease*”)) AND (TITLE-ABS-KEY (immunosuppressive OR immunosuppressant* OR corticosteroid* OR prednisone OR prednisolone OR mycophenolate OR tacrolimus OR cyclosporine)) AND (TITLE-ABS-KEY (randomized OR randomised OR “controlled trial” OR placebo OR randomly OR trial OR “clinical trial”))

6. China National Knowledge Infrastructure (CNKI)

主题=(雷公藤 OR 雷公藤多苷 OR 雷公藤总苷) AND 主题=(肾炎 OR 肾小球肾炎 OR 肾病综合征 OR 蛋白尿 OR 免疫性肾病 OR 肾脏疾病) AND 主题=(免疫抑制剂 OR 激素 OR 糖皮质激素 OR 泼尼松 OR 泼尼松龙 OR 霉酚酸酯 OR 他克莫司 OR 环孢素) AND 主题=(随机 OR 对照 OR 临床试验 OR 随机对照试验)

Subject=(tripterygium OR “tripterygium glycosides” OR “thunder god vine”)AND Subject=(“kidney disease” OR nephritis OR glomerulonephritis OR “nephrotic syndrome” OR proteinuria) AND Subject=(immunosuppressive OR corticosteroid OR prednisone OR mycophenolate OR tacrolimus) AND Subject=(randomized OR “controlled trial” OR “clinical trial”)

7. Wanfang Database

主题: (雷公藤+雷公藤多苷+雷公藤总苷) *主题: (肾病+肾炎+肾小球肾炎+肾病综合征+蛋白尿+免疫性肾病) * 主题: (免疫抑制剂+激素+糖皮质激素+泼尼松+泼尼松龙+霉酚酸酯+他克莫司+环孢素) * 主题: (随机+对照+临床试验+随机对照试验)

8. VIP Database (Chinese Scientific Journals Database)

M= (雷公藤 OR 雷公藤多苷 OR 雷公藤总苷) AND M= (肾病 OR 肾炎 OR 肾小球肾炎 OR 肾病综合征 OR 蛋白尿 OR 免疫性肾病 OR 肾脏疾病) AND M= (免疫抑制剂 OR 激素 OR 糖皮质激素 OR 泼尼松 OR 泼尼松龙 OR 霉酚酸酯 OR 他克莫司 OR 环孢素) AND M=(随机 OR 对照 OR 临床试验 OR 随机对照试验)

9. ClinicalTrials.gov

Condition or Disease: kidney disease OR renal disease OR glomerulonephritis OR nephrotic syndrome OR nephritis OR proteinuria.

Other Terms: tripterygium OR “tripterygium glycosides” OR “tripterygium wilfordii” OR “thunder god vin” OR “lei gong teng”.

Intervention/Treatment: immunosuppressive OR corticosteroid OR prednisone OR prednisolone OR mycophenolate OR tacrolimus OR cyclosporine.
